# Diarrhœas of Children

**Published:** 1878-12-20

**Authors:** 


### DIARRHŒAS OF CHILDREN.

In over one-half of the cases of diarrhœa that have come under my care during the last few years, pepsine has been the only medicine necessary; has been given after each movement, in 3 to 5 gr. doses, in milk, or in a mixture of glycerine. Dilute muriatic acid, cinnamon or winter-green water, or combined with bi-carb. sodæ, 2 grs., if there was much acidity of the secretions. If an astringent is necessary it may be added to the pepsine mixture. Generally 5 or 10 drops of the fl. ext. of blackberry root, or of the geranium maculatum, is sufficient for a dose. These astringents have seemed to me to be preferable to Kino, Catechu, etc.

The medicinal mist. rhei et sodæ has been used in about one-fourth of the cases where an astringent and alkali were needed. Generally but a few doses were needed when pepsine could be used.

Malarial diarrhœa is relieved by the inunction of 3 grs. of quinine twice or thrice a day till 12 grs. are used.

The hypodermic injection of 1-90 gr. of strychnia, p. r. n., in severe prostration, not otherwise amenable to treatment, is valuable.

One-drop doses of tr. or wine of ipecac, or a fraction of a drop of the fl. ext., or of ac. carbolic, given every hour, will ordinarily relieve the vomiting occurring with diarrhœas.

Aromatic spirits of ammonia seem to be a more reliable stimulant than alcohol.

Cod-liver oil, dialysed iron and the iodide of iron carefully given, *after* meals, beginning treatment with *small* doses, are serviceable in chronic diarrhœa.

Calomel, opiates, sedatives, or strong astringents were used in a small proportion of cases – less than one-eighth, and are seldom deemed necessary *if* the hygienic treatment can be carried out.

In closing this paper I may add that it was written as an outgrowth of a large experience in the treatment of diarrhœas; is a contribution of personal experience only, the result of what seems to me to be a better and more rational method of treatment than I was instructed in, in my college days. Certainly it has been attended in my hands by a larger proportion of recoveries than by old methods. The record of individual cases must be postponed to another time.—*Dr. Walker, in Soc. County of Kings.*

